# Early response and pathological complete remission in Breast Cancer with different molecular subtypes: a retrospective single center analysis

**DOI:** 10.7150/jca.46805

**Published:** 2020-10-06

**Authors:** Jin Hong, Jiayi Wu, Ou Huang, Jianrong He, Li Zhu, Weiguo Chen, Yafen Li, Xiaosong Chen, Kunwei Shen

**Affiliations:** Department of General Surgery, Comprehensive Breast Health Center, Ruijin Hospital, Shanghai Jiao Tong University School of Medicine, Shanghai, 200025, PR China.

**Keywords:** Breast cancer, clinical early response, pathological complete response, neoadjuvant treatment

## Abstract

**Purpose:** To evaluate the association of clinical early response and pathological complete remission (pCR) in breast cancer patients with different molecular subtypes.

**Materials and methods:** Breast cancer patients who received neoadjuvant treatment (NAT) with clinical early response assessment from October 2008 to October 2018 were retrospectively analyzed. Clinical early response was defined as tumor size decreasing ≥30% evaluated by ultrasound after two cycles of NAT. Chi-square test was used to compare the pCR rates between the responder and non-responder groups with different molecular subtypes. Multivariate logistic regression was used to identify independent factors associated with the pCR.

**Results:** A total of 328 patients were included: 100 responders and 228 non-responders. The progesterone receptor (PR) expression was an independent factor associated with clinical early response (OR=2.39, 95%CI=1.41-4.05, *P*=0.001). The pCR rate of breast was 50.0% for responders and 18.0% for non-responders (*P*<0.001). Regarding different molecular subtypes, responders had higher pCR rates than non-responders for patients with HER2 overexpression (OR=10.66, 95%CI=2.18-52.15, *P*=0.001), triple negative (OR=3.29, 95%CI=1.23-8.84, *P*=0.016) and Luminal (HER2-) subtypes (OR=8.58, 95%CI=3.05-24.10, *P*<0.001) respectively. Moreover, pCR rate can be achieved as high as 88.2% in HER2 overexpression patients with early clinical response, which was significantly higher than patients without early response (41.3%, *P*=0.001). Multivariate analysis showed that clinical early response was an independent factor associated with the pCR rate (OR=4.87, 95%CI=2.72-8.72, *P*<0.001).

**Conclusions:** Early response was significantly associated with a higher pCR rate in breast cancer patients receiving NAT, especially for patients with HER2 overexpression subtype, which warrants further clinical evaluation.

## Introduction

Neoadjuvant treatment (NAT) is a common strategy to downstage locally advanced diseases and increase breast conservative surgery rate in breast cancer patients 1, 2, which was found to have similar disease outcome compared to patients receiving adjuvant chemotherapy 3, 4. Nevertheless, patients who had achieved pathological complete remission (pCR) after NAT had significantly better disease free survival (DFS) and overall survival (OS) than those without pCR 5, 6, which will accelerate drug efficacy evaluation and new drug approval.

As a result, achieving pCR becomes quite an important aim for better outcomes in NAT. The reported pCR rates varied with different phenotypes from 0% to 79% in previous studies 6. The tumor biology is a critical factor associated with pCR after NAT, estrogen receptor (ER) and progesterone receptor (PR) negativity, human epidermal growth factor receptor-2 (HER2) positivity, high mitotic count and Ki67 score were correlated with pCR^6^, ^7^. A retrospective study from Memorial Sloan Kettering Cancer Center (MSKCC) found that patients with HER2 positive tumors had the highest pCR rate and the lowest pCR rate appeared in the hormone receptor (HR) positive HER2 negative subgroup 8.

Therapeutic response differs individually in breast cancer patients and clinical response is a method for early evaluation. Aside from molecular markers, clinical response to initial cycles of neoadjuvant chemotherapy appeared to be associated with the pCR rate 9. Therefore, early identification of clinical response in neoadjuvant therapy may help us to select those who are responsive to initial regimens. For non-responders, it may provide an opportunity to get an alternative therapy. Few studies evaluated the predictive value of early response assessed by ultrasound for pCR in different breast cancer molecular subtypes and survival. In this study, we aimed to evaluate the association of clinical early response and pCR rate in breast cancer patients with different molecular subtypes as well as survival outcome.

## Materials and Methods

### Patient population

**Figure 1:**
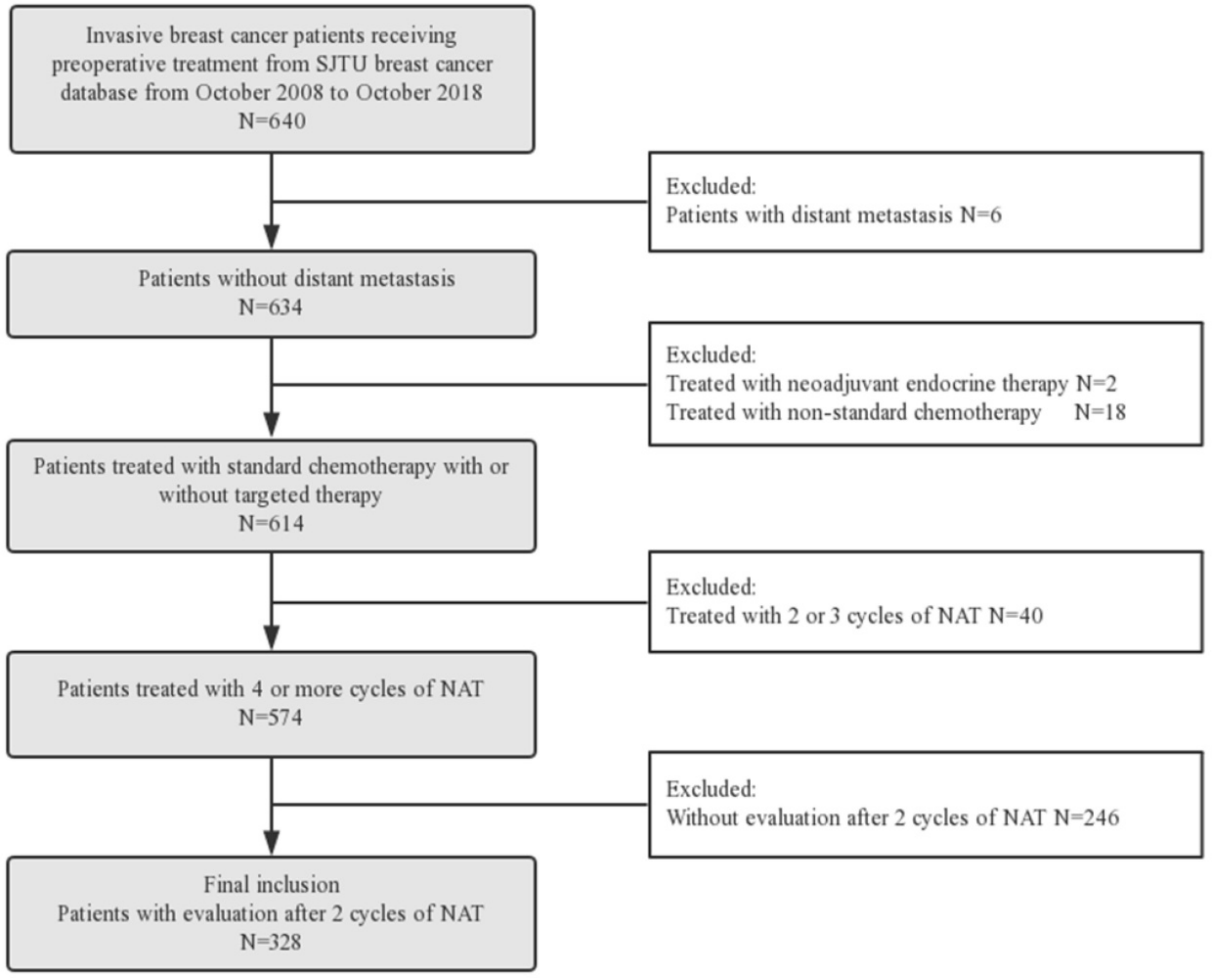
** Patient Flow Diagram of the study.** Abbreviation: NAT: neoadjuvant treatment.

Six hundred and forty consecutive breast cancer patients who received preoperative treatment from October 2008 to October 2018 in Shanghai Jiao Tong University Breast Cancer Database (SJTU-BCDB) were analyzed retrospectively (Figure 1). Eligibility criteria were as follows: (1) invasive breast cancer; (2) received standard neoadjuvant chemotherapy and/or trastuzumab for ≥ 4 cycles; (3) with clinical response assessment after two cycles of NAT; (4) with pathological evaluation after surgery. Patients with following criteria were excluded: (1) with distant metastasis; (2) received neoadjuvant endocrine therapy or non-standard chemotherapy (capecitabine or vinorelbine); (3) received NAT < 4 cycles; (4) without clinical response assessment after two cycles of NAT.

All patients received neoadjuvant chemotherapy and patients with HER-2 positive could also receive trastuzumab. Anthracycline (A) and taxanes (T) based regimens included EC-T (epirubicin 90 mg/m^2^, cyclophosphamide 600 mg/m^2^ and followed by docetaxel 100 mg/m^2^) and TEC (docetaxel 75 mg/m^2^, epirubicin 75 mg/m^2^ and cyclophosphamide 500 mg/m^2^) every 21 days. Anthracycline or taxanes based regimens included EC (epirubicin 90 mg/m^2^ and cyclophosphamide 600 mg/m^2^) or TC (docetaxel 75 mg/m^2^ and cyclophosphamide 600 mg/m^2^) every 21 days. Trastuzumab(H) was administrated 8 mg/Kg at first cycle and followed by 6 mg/Kg every 3 weeks or 4 mg/Kg at first cycle and followed by 2 mg/Kg every week. Targeted combined with chemotherapy regimens included EC-TH, TCbH (docetaxel 75 mg/m^2^ and carboplatin AUC 6) every 21 days or PCbH (paclitaxel 80 mg/m^2^ and carboplatin AUC 2) every 7 days.

### Clinical and pathological evaluation

Baseline patients' characteristics were obtained from the SJTU-BCDB, including age, menopausal status, clinical tumor stage, clinical nodal stage and neoadjuvant treatment cycles. Pathological characteristics were confirmed by core needle biopsy, including pathology type, tumor grade, ER, PR and C-erbB-2. ER and PR positivity were defined as more than 1% positive invasive tumor cells with nuclear staining. Tumors with immunohistochemical (IHC) HER-2 3+ and/or HER2 gene amplification confirmed by florescent in situ hybridization (FISH) were regarded as HER2 positive. Tumors were classified as four molecular subtypes: Luminal-HER2 negative (HR+HER-), Luminal-HER2 positive (HR+HER2+), HER2 overexpression (HR-HER2+) and triple negative breast cancer (TNBC, HR-HER2-).

Clinical response assessment was performed by three-dimensional (3D) ultrasound before NAT and after two cycles of NAT. The tumor response can be classified into four types: complete response (CR, disappearance of all target lesions), partial response (PR, at least a 30% decrease in the sum of diameters of target lesions), progressive disease (PD, at least a 20% increase in the sum of diameters of target lesions) and stable disease (SD, neither PD nor PR) according to the Response Evaluation Criteria in Solid Tumor (RECIST) version 1.1^10^. Patients with CR or PR after two cycles of NAT were defined as responders and patients with PD or SD were defined as non-responders. Pathological response was evaluated after surgery. pCR in breast was defined as absence of invasive cancer in the breast, irrespective of DCIS or nodal involvement (ypT0/is) 11.

### Statistical analysis

Categorical variables between two groups were compared using two-sided Pearson chi-square test. Multivariable logistic regression model was used to determine the independent predictive factor for pCR and variables with *p* value <0.05 in the univariate analysis were included. DFS was defined as the interval from the day of surgery to the date of any event as follows: any local or regional recurrence, distant metastasis, newly diagnosed contralateral breast cancer, any secondary malignancy or death from any cause. OS was defined as the interval from the day of surgery to the date of death from any cause. DFS and OS were analyzed and compared using Kaplan-Meier method and the log-rank test. Statistical analyses were performed using SPSS (version 22.0) software (IBM Corporation, Armonk, NY, USA).

## Results

### Patients' characteristics

Between October 2008 and October 2018, 640 invasive breast cancer patients receiving preoperative treatment were collected from SJTU-BCDB. Among them, 328 patients who were treated with 4 or more cycles of NAT and assessed for clinical early response by 3D ultrasound were included in the subsequent analyses (Figure 1). Among those patients, 100 were responders and 228 were non-responders.

Patients' characteristics were summarized in Table 1. The median age was 55 years old and 50% of the patients were premenopausal women. 263 patients had cT0 to cT2 disease and 65 patients had cT3 /4 disease. 220 patients were diagnosed as cN0 or cN1 and 101 patients had cN2/3 disease. Among those patients, 138 patients were Luminal (HER2-) subtype, 53 patients were Luminal (HER2+) subtype, 63 patients were HER2 overexpression and 74 patients were triple negative breast cancer. 254 patients received anthracycline and taxanes (A+T) combined neoadjuvant chemotherapy, and 74 patients received anthracycline (A) or taxanes (T) based regimens. 86 of 116 HER-2 positive patients received trastuzumab. Among those patients, 76 patients received only 4 cycles of NAT and 252 patients received more than 4 cycles of treatment.

### Factors associated with clinical early response

In the univariate analysis, the proportion of responders was significantly higher in ER negative tumors than ER positive tumors (36.5% vs 26.2%, *P* = 0.045) and in PR negative tumors than PR positive tumors (36.7% vs 19.7%, *P* = 0.001) (Table 1). The highest proportion of responders was 44.6% in the TNBC subgroup and the lowest was 24.6% in the Luminal (HER2-) group (*P* = 0.023). Multivariate analysis indicated PR was an independent factor for clinical early response (OR = 2.39, 95%CI = 1.41-4.05, *P* = 0.001) (Table 2).

### Clinical early response and the pCR rate

**Figure 2:**
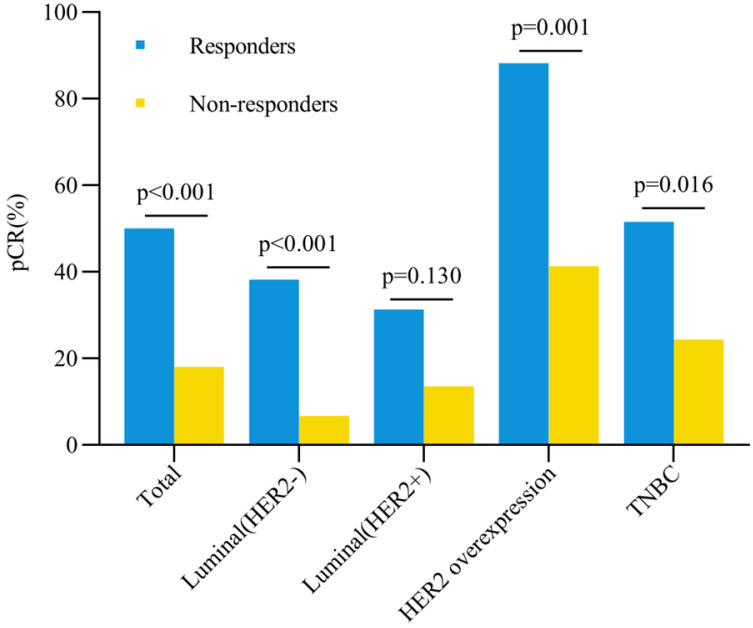
** Breast pCR rate and early clinical response.** Abbreviation: pCR: pathological complete response, HER2: human epidermal growth factor receptor-2, TNBC: triple negative breast cancer.

The total pCR rate in breast was 50% for responders and 18% for non-responders (*P* < 0.001) (Figure 2). Univariate analysis showed that clinical early response, ER expression, PR expression, HER-2 status and targeted therapy were correlated with pCR rates after NAT (all *P* < 0.05, Table 3). Logistic regression multivariate analysis revealed that clinical early response was an independent predictive factor for pCR in breast (OR = 4.87, 95%CI = 2.72-8.72, *P* < 0.001) (Table 4). In addition, other factors were also associated with pCR in breast significantly, including ER status (OR = 2.20, 95%CI = 1.12-4.34, *P* = 0.022), PR status (OR = 2.68, 95%CI = 1.13-6.33, *P* = 0.025) and targeted therapy (OR = 2.41, 95%CI = 1.31-4.44, *P* = 0.005) (Table 4).

**Figure 3:**
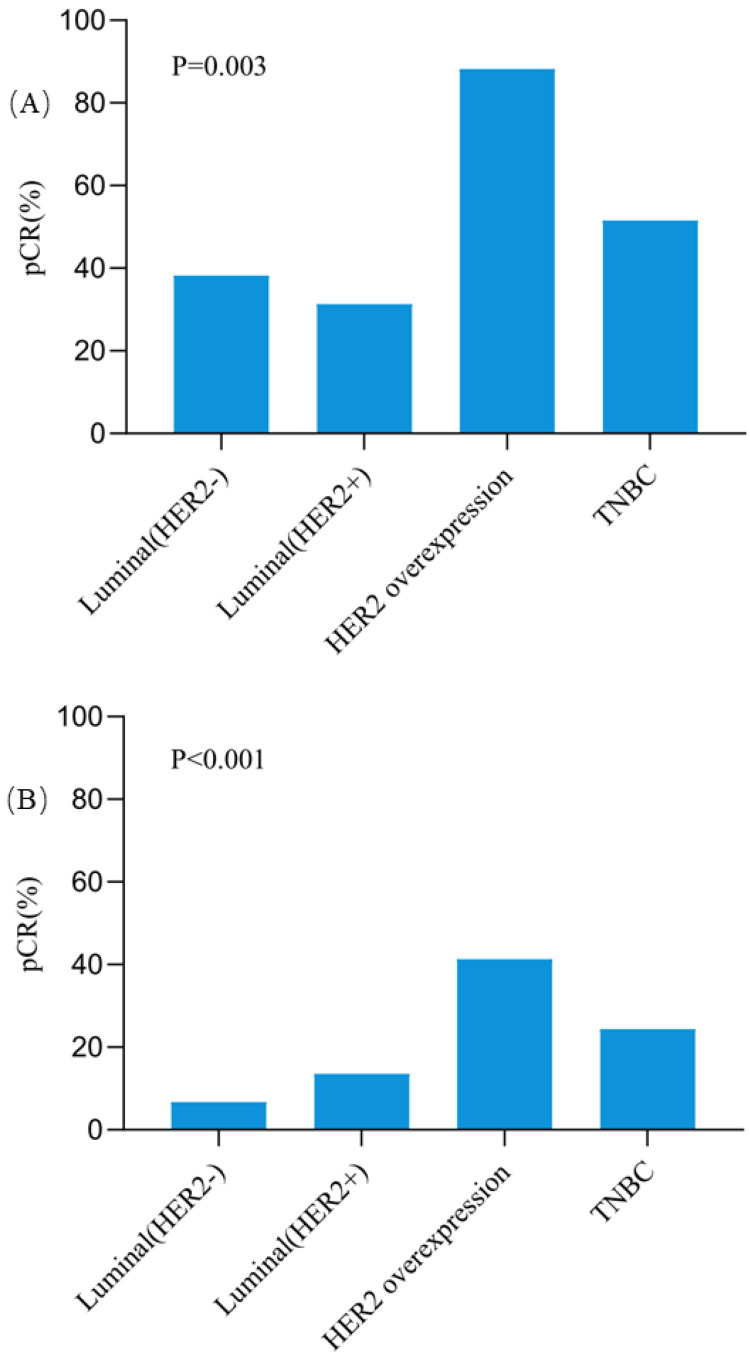
** The pCR rates in Breast among different molecular subtypes in clinically early responders (A) and non-responders (B).** Abbreviation: *pCR:* pathological complete response, HER2: human epidermal growth factor receptor-2, TNBC: triple negative breast cancer.

The pCR rates in breast for different subtypes were also further analyzed (Figure 2). Except the Luminal (HER2+) tumors, all three other subtypes showed higher pCR rates in responders than non-responders. In patients with HER2 overexpression tumors, the pCR rate was as high as 88.2% for responders, which was significantly higher than 41.3% for non-responders (OR = 10.66, 95%CI = 2.18-52.15, *P* = 0.001). In triple negative breast cancer patients, responders also had a significantly higher pCR rate than non-responders (51.5% vs 24.4%, OR = 3.29, 95%CI = 1.23-8.84, *P* = 0.016). Among patients with Luminal (HER2-) tumors, the pCR rate was 38.2% for responders and 6.7% for non-responders (OR = 8.58, 95%CI = 3.05-24.10, *P* < 0.001). However, no significant difference was observed for pCR between responders and non-responders in Luminal (HER2+) patients (31.3% vs 13.5%, OR = 2.90, 95%CI = 0.71-12.00, *P* = 0.130). Besides, compared with other subtypes, patients with HER2 overexpression achieved the highest pCR rate both in responders (*P* = 0.003, Figure 3A) and non-responders (*P* < 0.001, Figure 3B).

### Clinical early response and disease survival

**Figure 4:**
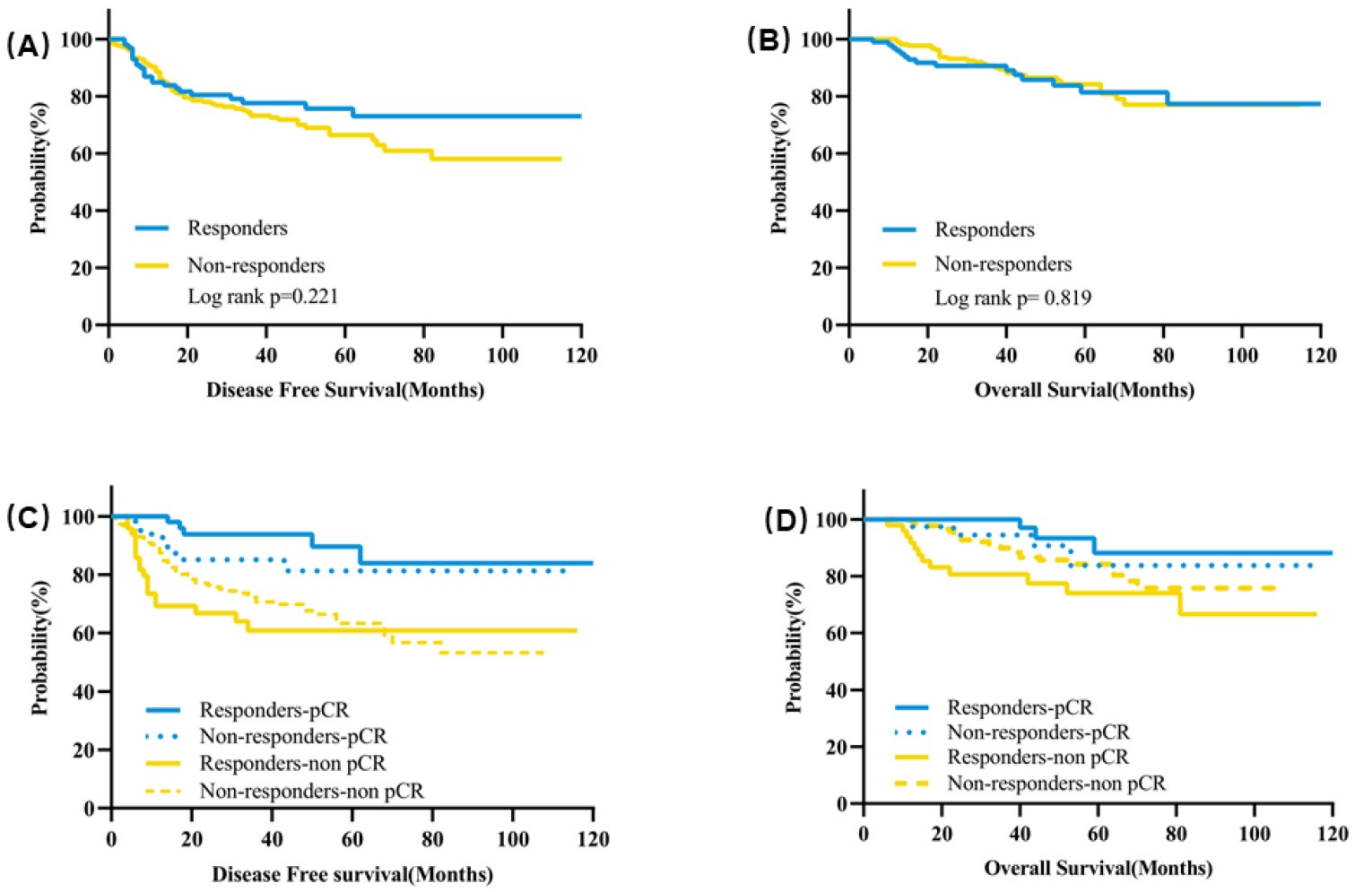
** Kaplan-Meier curves of survival for clinical responders and non-responders after two cycles of NAT.** (**A**) DFS according to clinical early response; (**B**) OS according to clinical early response; (**C**) DFS according to clinical early response and pathological response after NAT; (**D**) OS according to clinical early response and pathological response after NAT. Abbreviation: DFS: disease free survival, OS: overall survival, NAT: neoadjuvant treatment.

The median follow-up time was 43 months. The estimated 5-year DFS was 75.6% for responders and 66.4% for non-responders (*P* = 0.221) (Figure 4A). There was also no significant difference for the estimated 5-year OS between responders and non-responders (81.4% vs 84.2%, *P* = 0.819) (Figure 4B). In terms of subgroup analysis according to pathological response, no significant differences were observed for the 5-year DFS or OS between responders and non-responders neither in the patients with pCR nor in patients with residual tumors (Figure 4C&D, Table S1).

In responders, the estimated 5-year DFS was significantly higher for patients with pCR than patients not achieving pCR (89.6% vs 60.9%, *P* = 0.0008) (Figure 3C, Table S2). Patients achieving pCR also had a significantly higher estimated 5-year OS than patients with residual tumors (93.3% vs 74.0%, *P* = 0.010) (Figure 4D, Table S2). In non-responders, the estimated 5-year DFS was 81.2% for patients with pCR compared with 62.5% for patients with residual tumor (*P* = 0.066) and the estimated 5-year OS was 83.7% for patients with pCR and 84.2% for patients without pCR (*P* = 0.405) (Figure 4C&D, Table S2).

## Discussion

Clinical early response to NAT was an excellent predictor for pCR in breast. Our current study showed that early response was significantly associated with a higher pCR rate in breast cancer patients receiving NAT, especially for patients with HER2 overexpression subtype.

Neoadjuvant therapy is the preferred treatment of locally advanced breast cancer for the purpose of creating surgery opportunities 12. An increasing number of operable breast cancer patients also receive NAT, which provides us an opportunity to observe the tumor shrink and response to neoadjuvant regimens, especially in the clinical trial for new drug development 1. In the NeoSphere trial, the addition of pertuzumab to trastuzumab and docetaxel resulted in significantly higher pCR rates 13, accelerating the approval of pertuzumab in the neoadjuvant treatment of HER2 positive breast cancer by the Food and Drug Administration (FDA) 14. Although the overall survival benefit of NAT remained controversial and even a higher local recurrence rate after breast conserving surgery was found in patients receiving NAT than those treated in the adjuvant setting 4, patients achieving pCR after NAT had better DFS and OS than patients with residual tumors 5, 6. Our study also confirmed that patients with pCR after NAT had significantly better DFS and OS than patients not achieving pCR in the responder group.

To date, three main methods were reported for early response assessment in neoadjuvant treatment of breast cancer: ^18^F-FDG PET/CT, dynamic contrast enhanced MRI (DCE-MRI), and ultrasound 9, 15, 16. Meta-analysis showed that ^18^F-FDG PET/CT had a moderate accuracy for the early prediction for pCR (sensitivity 85%, specificity 79% and diagnostic odds ratio 21.8), especially in HER2 positive and triple negative breast cancer 16, 17. DCE-MRI was also an effective method for early monitoring the efficacy during NAT (sensitivity 87%, specificity 82% and diagnostic odds ratio 30.3) 18. However, both ^18^F-FDG PET/CT and DCE-MRI require sophisticated imaging, additional radiation dose for patients, and less accessible compared with ultrasound. A systemic search in PubMed was performed, nine studies on the early prediction for pathological response by ultrasound in breast cancer are summarized in Table S4 9, 19-26. The diagnostic accuracy for pathological response with different parameters assessed by ultrasound during NAT was performed in eight studies and showed moderate results with different sensitivity and specificity. Among these studies, only two had estimated pCR rate different between early responders and non-responders, which were also not analyzed and compared among different subtypes 9, 19.

The pCR rates varied among different molecular subtypes. In a retrospective study with 13,939 breast cancer patients, patients with the Luminal A subtype had the lowest pCR rate (0.3%) and the highest pCR rate was observed in HER-2 positive patients (38.7%) 27. Most of previous studies supported that patients with HER-2 positive breast cancer who received chemotherapy combination with targeted therapy achieved the highest pCR rate, especially those treated with dual targeted therapy 8, 27, 28. The total clinical response rate was 30.5% in our study and responders had a significant higher pCR rate than non-responders (50% vs 18%). To our knowledge, no previous studies had ever focused on the predictive value of clinical early response for pCR in different molecular subtypes. In our study, its predictive value was largely independent of the molecular subtypes as except for Luminal (HER2+) group, responders had significantly higher pCR rates than non-responders in other three subgroups. The highest pCR rate was 54% in HER2 overexpression patients and responders achieved a pCR rate of up to 88.2%, which was analogous to patients receiving dual HER-2 blockade in combination with chemotherapy reported previously 13, 29-32. The unexpected results indicated that patients with HER2 overexpression in breast cancer and have excellent early clinical response to NAT with chemotherapy and trastuzumab, do not need escalation of dual anti-HER2 therapy. However, we also found that the Luminal (HER2+) subset was not benefit from clinical early response. Previous trials had showed that the pCR rates were higher in HER2+/HR- patients than HER2+/HR+ patients 32, 33. Von Minckwitz and colleges also demonstrated that pCR was a surrogate end point for patients with triple negative, Luminal B/HER2-, and HER2 positive breast cancer but not for patients with Luminal B/HER2+ disease 34. Therefore, the Luminal (HER2+) breast cancer was a special subtype that needed further clinical trials exploring novel systemic treatments. We also analyzed the pCR in breast and node (ypT0/isN0) and found the similar results in the whole population and different molecular subsets (Figure S1). Neoadjuvant regimens and treatment cycles may also influence treatment response 5, 35. However, there were no differences for pCR rates between patients receiving different chemotherapy regimens or different NAT cycles in our study. While patients treated with trastuzumab had significantly higher pCR rates than those without trastuzumab treatment (Tables 3 and 4).

A previous study had also explored the predictive value of clinical response after two cycles of chemotherapy for pCR (*P* = 0.003), in which responders included patients who had a 50% or greater reduction in tumor measurements 9. In our study, however, responders were defined as patients with at least a 30% decrease of tumor size. The predictive value of different cut-off points of tumor size reduction (TSR) after two cycles of NAT had also been explored (Figure S2). Patients with TSR ≥ 20% had a higher pCR rate than patients with TSR < 20% (Breast: 43.1% vs 15.8%, *P* < 0.001; Breast and Node: 27.8% vs 10.9%, *P* < 0.001). If responders were defined as patients with TSR ≥ 10%, responders still had a significant higher pCR rate than non-responders (Breast: 37.6% vs 14.4%, *P* < 0.001; Breast and Node: 24.9% vs 9.4%, *P* < 0.001). More importantly, the pCR rate deceased with the lower of the cut-off point of TSR and 30% was an appropriate cut-off point for TSR in our study.

As clinical early response to NAT is correlated with pCR, we analyzed factors associated with clinical early response and found that PR was an independent predictive factor. Patients with PR negative tumors had a 2.39 fold possibility to achieve early response than patients with PR positive disease. The prognostic value of clinical early response was also explored in our study and clinical early response could not translate into survival benefits. No significant differences were observed for DFS and OS between responders and non-responders irrespective of pathological response status. Meanwhile, in clinical responders, patients with pCR had significantly better DFS and OS than patients with residual tumors. Regarding non-responders, patients achieving pCR had a numerically higher DFS than patients with non-pCR, although there was no significant difference. Hence, pathological response can provide additional prognostic value for survival beyond clinical early response and clinical responders achieving pCR after NAT gained the greatest survival benefit. Moreover, what is the interference for non-responders? In the GeparTrio trial, after two cycles of TAC (docetaxel, doxorubicin and cyclophosphamide), early non-responders were randomly assigned to an additional 4 cycles of TAC or to 4 cycles of NX (vinorelbine and capecitabine), the clinical response and pCR rates were similar between two groups^36^. However, DFS was higher in patients receiving TAC-NX than 6 cycles of TAC, which was mainly reflected in HR positive patients 37. This implied that non-responders after two cycles of NAT could receive an alternative regimen for better outcome.

There were some limitations of the study. Firstly, it was a retrospective study and neoadjuvant treatment regimens and cycles were diverse across patients. Secondly, as a single center analysis, patient numbers included in our study was not so large, especially for subgroup analysis. Thirdly, we could not validate our findings and conclusions with an independent cohort.

## Conclusions

In conclusion, clinical responders after two cycles of NAT had a significantly higher pCR rate than non-responders. The predictive value of clinical early response was mainly observed in HER2 overexpression, triple negative, and Luminal (HER2-) breast cancer patients. Particularly, responders with HER2 overexpression subtype treated with trastuzumab had the highest pCR rate, which was analogous to those receiving dual HER2 blockade in previous literatures. Clinical early response after NAT may serve as an excellent marker for clinical decision making, deserving further clinical validation.

## Supplementary Material

Supplementary figures and tables.

## Figures and Tables

**Table 1:** Baseline Characteristics and Treatments

Characteristics	Total N=328 (%)	Responders N=100 (%)	Non-responders N=228 (%)	*P*-value
**Age, years**				0.491
<50	157 (47.9)	45 (28.7)	112 (71.3)	
≥50	171 (52.1)	55 (32.2)	116 (67.8)	
**Menopausal status**				1.000
Premenopausal	164 (50)	50 (30.5)	114 (69.5)	
Postmenopausal	164 (50)	50 (30.5)	114 (69.5)	
**Pathology**				0.509
IDC	272 (82.9)	85 (31.2)	187 (68.8)	
Others	56 (17.1)	15 (26.8)	41 (73.2)	
**Clinical T stage**				0.147
T0-2	263 (80.2)	85 (32.3)	178 (67.7)	
T3-4	65 (19.8)	15 (23.1)	50 (76.9)	
**Clinical N stage**				0.409
N0-1	220 (68.5)	64 (29.1)	156 (70.9)	
N2-3	101 (31.5)	34 (33.7)	67 (66.3)	
**Grade**				0.674
I-II	78 (23.8)	21 (26.9)	57 (73.1)	
III	144 (43.9)	44 (30.6)	100 (69.4)	
NA	106 (32.3)	35 (33.0)	71 (67.0)	
**ER**				0.045
positive	191 (58.2)	50 (26.2)	141 (73.8)	
negative	137 (41.8)	50 (36.5)	87 (63.5)	
**PR**				0.001
positive	122 (37.2)	24 (19.7)	98 (80.3)	
negative	206 (62.8)	76 (36.9)	130 (63.1)	
**HER-2**				0.553
positive	116 (35.4)	33 (28.4)	83 (71.6)	
negative	212 (64.6)	67 (31.6)	145 (68.4)	
**Subtype**				0.023
Luminal (HER2-)	138 (42.1)	34 (24.6)	104 (75.4)	
Luminal (HER2+)	53 (16.2)	16 (30.2)	37 (69.8)	
HER2 overexpression	63 (19.2)	17 (27.0)	46 (73.0)	
TNBC	74 (22.6)	33 (44.6)	41 (55.4)	
**Regimens**				0.065
A or T	74 (22.6)	29 (39.2)	45 (60.8)	
A and T	254 (77.4)	71 (28.0)	183 (72.0)	
**Target therapy**				0.250
Yes	86 (26.2)	22 (25.6)	64 (74.4)	
No	242 (73.8)	78 (32.2)	164 (67.8)	
**Neoadjuvant cycles**				0.814
4	76 (23.2)	24 (31.6)	52 (68.4)	
> 4	252 (76.8)	76 (30.2)	176 (69.8)	

Abbreviation: IDC: invasive ductal carcinoma, NA: not available, BCS: breast conserving surgery, ER: estrogen receptor, PR: progestrone receptor, HER2: human epidermal growth factor receptor-2, TNBC: triple negative breast cancer, A: anthracycline, T: taxanes.

**Table 2:** Multivariate analysis for factors associated with clinical early response

Characteristics	OR	95%CI	*P*-value
**ER status**			
positive	1		
negative	1.37	0.66-2.86	0.379
**PR status**			
positive	1		
negative	2.39	1.41-4.05	0.001
**Subtypes**			0.502
Luminal (HER2-)	1		
Luminal (HER2+)	0.93	0.45-1.93	0.841
HER2 overexpression	1.59	0.71-3.53	0.257
TNBC	0.73	0.35-1.52	0.397

Abbreviation:* ER* estrogen receptor, *PR* progestrone receptor, *HER2* human epidermal growth factor receptor-2, *TNBC* triple negative breast cancer, *OR* odds ratio, *CI* confidence interval.

**Table 3:** Clinicopathological factors and pCR in Breast

Characteristics	pCR (No.)	Non-pCR (No.)	*P*-value
**Age**			0.890
<50	43	114	
≥50	48	123	
**Pathology**			0.879
IDC	75	197	
Others	16	40	
**Clinical T stage**			0.062
T0-2	79	184	
T3-4	12	53	
**Clinical N stage**			0.653
N0-1	60	160	
N2-3	30	71	
**ER status**			<0.001
positive	30	161	
negative	61	76	
**PR status**			<0.001
positive	11	111	
negative	80	126	
**HER2 status**			0.002
positive	44	72	
negative	47	165	
**Regimen**			0.306
A or T	24	50	
A and T	67	187	
**Target therapy**			<0.001
Yes	37	49	
No	54	188	
**Neoadjuvant Cycles**			0.751
4	20	56	
> 4	71	181	
**Clinical early response**			<0.001
Responders	50	50	
Non-responders	41	187	

Abbreviation: ER estrogen receptor, PR progestrone receptor, HER2 human epidermal growth factor receptor-2, A anthracycline, T taxanes.

**Table 4:** Multivariate analysis of factors associated with pCR in Breast

Characteristics	OR	95%CI	*p*-value
**ER status**			
positive	1		
negative	2.20	1.12-4.34	0.022
**PR status**			
positive	1		
negative	2.68	1.13-6.33	0.025
**HER2 status**			
negative	1		
positive	1.14	0.48-2.71	0.768
**Target therapy**			
No	1		
Yes	2.41	1.31-4.44	0.005
**Clinical early response**			
Non-responders	1		
Responders	4.87	2.72-8.72	<0.001

Abbreviation: ER: estrogen receptor, PR: progestrone receptor, HER2: human epidermal growth factor receptor-2, OR: odds ratio, CI: confidence interval, NAT: neoadjuvant treatment.
