# Supplementary material for: Early response and pathological complete remission in Breast Cancer with different molecular subtypes: a retrospective single center analysis
Source: J Cancer. 2020 Oct 6;11(23):6916–24. doi: 10.7150/jca.46805 (PMC7591996; doi:10.7150/jca.46805)
Supplement: Supplementary file 1 — Supplementary figures and tables. [file jcav11p6916s1.pdf]

Table S1. Comparison of 5-year DFS and OS between responders and non-responders according to pathological response.

Pathological response	Clinical response	5-year DFS	*P*-value	5-year OS	*P*-value
pCR	Responders	89.6%	0.244	93.3%	0.405
	Non-responders	81.2%		83.7%	
Non-pCR	Responders	60.9%	0.437	74.0%	0.090
	Non-responders	62.5%		84.2%	

Abbreviation: *pCR* pathological complete response, *DFS* disease free survival, *OS* overall survival, *NAT* neoadjuvant treatment.

Table S2. Comparison of 5-year DFS and OS between patients with pCR and non pCR after NAT according to clinical early response.

Clinical response	Pathological response	5-year DFS	*P*-value	5-year OS	*P*-value
Responders	pCR	89.6%	0.0008	93.3%	0.010
	Non-pCR	60.9%		74.0%	
Non-responders	pCR	81.2%	0.066	83.7%	0.521
	Non-pCR	62.5%		84.2%	

Abbreviation: *pCR* pathological complete response, *DFS* disease free survival, *OS* overall survival, *NAT* neoadjuvant treatment.

Table S3 Comparison of the pCR rates in breast among different molecular subtypes.

**Molecular subtypes**	**Luminal (HER2-)**	**Luminal (HER2+)**	**HER2 overexpression**	**TNBC**	**P-value**
**Total**	14.5%	18.9%	54.0%	36.5%	**<0.001**
**Responder**	38.2%	31.3%	88.2%	51.5%	**0.003**
**Non-responder**	6.7%	13.5%	41.3%	24.4%	**<0.001**

Abbreviation: *pCR* pathological complete response, *HER2* human epidermal growth factor receptor-2, *TNBC* triple negative breast cancer.

Table S4 Overview of studies about early assessment by ultrasound and pathological response in breast cancer patients receiving neoadjuvant therapy.

Study(year)	No. of patient	Breast subtypes	Timing of assessment	Parameter tested	Response standard	End point	Sensitivity	Specificity	pCR in responder	pCR in non-responder
Beresford MJ (2008) [3]	200	NR	Two cycles	Bi-dimensional	≥50% reduction	pCR	95.2%	52.9%	21.5%	1.2%
Cosimo SD (2018) [8]	340	HER2 positive	Two cycles	The longest diameter	≥30% reduction	pCR	NR	NR	46%	23%
Marinovich ML (2014) [9]	832	Whole	Two cycles	Bi-dimensional	≥50% reduction	pCR	81.7%	47.6%	NR	NR
Fernandes J (2019) [10]	92	Whole	4 weeks	Strain elastography	NR	pCR	85%	83%	NR	NR
Lee YJ (2018) [11]	41	NR	One cycle	Time-intensity curve	NR	M-P score 4 or 5	77%	67%	NR	NR
Wang XG (2016) [2]	290	NR	Two cycles	LDC and PC	≥50% reduction	pCR	88.1%	85.1%	NR	NR

Jing H (2016) [12]	62	NR	Two cycles	Shear wave elastography	NR	M-P score 4 or 5	72.9%	85.7%	NR	NR
Sannachi L (2015) [13]	30	NR	4 weeks	Backscatter parameters	NR	Response	82%	100%	NR	NR
Falou O (2013) [14]	15	NR	4 weeks	Elastographic parameters	NR	Response	100%	100%	NR	NR

Abbreviations: pCR, pathological complete response; HER2, human epidermal growth factor receptor-2; NR, not reported; M-P, Miller Payne; LDC, the longest diameter change; PC, the largest perpendicular diameter.

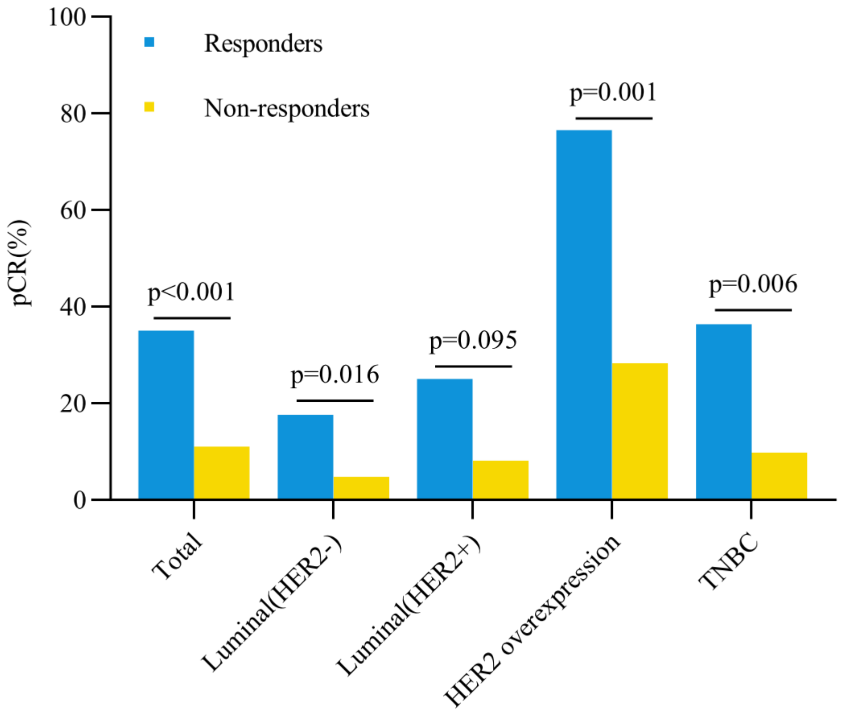

Figure S1 The pCR rate in Breast and node for clinical responders and non-responders after two cycles of neoadjuvant treatment.

Abbreviation: *pCR* pathological complete response, *HER2* human epidermal growth factor receptor-2, *TNBC* triple negative breast cancer.

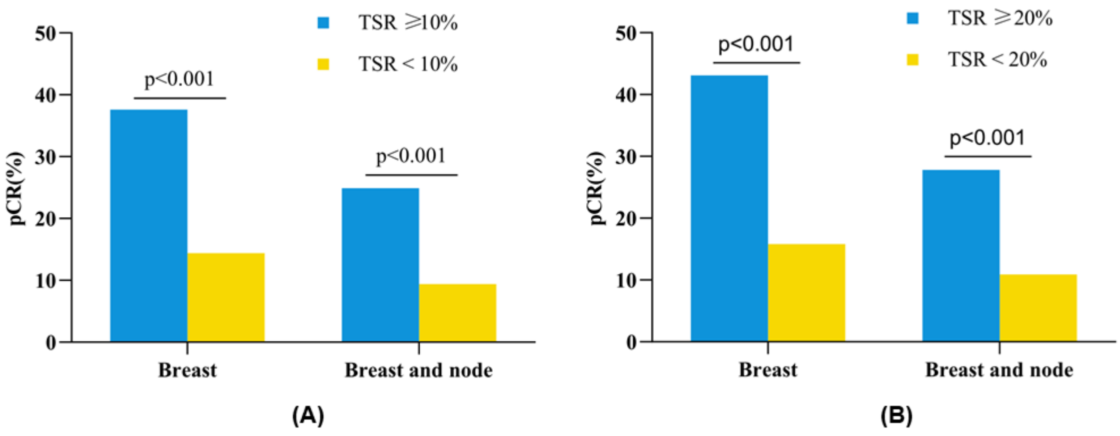

Figure S2 The pCR rate (Breast) and pCR rate (Breast and node) for patients with TSR ≥10% and TSR<10% (A) and TSR ≥20% and TSR<20% (B) after two cycles of neoadjuvant treatment.

Abbreviations: *pCR* pathological complete response, *TSR* tumor size reduction.
